# Contamination of U.S. Butter with Polybrominated Diphenyl Ethers from Wrapping Paper

**DOI:** 10.1289/ehp.1002604

**Published:** 2010-12-07

**Authors:** Arnold Schecter, Sarah Smith, Justin Colacino, Noor Malik, Matthias Opel, Olaf Paepke, Linda Birnbaum

**Affiliations:** 1 University of Texas School of Public Health, Dallas, Texas, USA; 2 University of Michigan School of Public Health, Ann Arbor, Michigan, USA; 3 Eurofins GfA, GmbH, Hamburg, Germany; 4 National Institute of Environmental Sciences/National Cancer Institute/National Institutes of Health, Department of Health and Human Resources, Research Triangle Park, North Carolina, USA

**Keywords:** butter, dietary intake, food, PBDEs, United States

## Abstract

**Objectives:**

Our aim was to report the first known incidence of U.S. butter contamination with extremely high levels of polybrominated diphenyl ethers (PBDEs).

**Methods:**

Ten butter samples were individually analyzed for PBDEs. One of the samples and its paper wrapper contained very high levels of higher-brominated PBDEs. Dietary estimates were calculated using the 2007 U.S. Department of Agriculture Loss-Adjusted Food Availability data, excluding the elevated sample.

**Results:**

The highly contaminated butter sample had a total upper bound PBDE level of 42,252 pg/g wet weight (ww). Levels of brominated diphenyl ether (BDE)-206, -207, and -209 were 2,000, 2,290, and 37,600 pg/g ww, respectively. Its wrapping paper contained a total upper-bound PBDE concentration of 804,751 pg/g ww, with levels of BDE-206, -207, and -209 of 51,000, 11,700, and 614,000 pg/g, respectively. Total PBDE levels in the remaining nine butter samples ranged from 180 to 1,212 pg/g, with geometric mean of 483 and median of 284 pg/g. Excluding the outlier, total PBDE daily intake from all food was 22,764 pg/day, lower than some previous U.S. dietary intake estimates.

**Conclusion:**

Higher-brominated PBDE congeners were likely transferred from contaminated wrapping paper to butter. A larger representative survey may help determine how frequently PBDE contamination occurs. Sampling at various stages in food production may identify contamination sources and reduce risk.

Some episodes of food contamination with persistent organic pollutants (POPs) are well known, such as the Yusho and Yucheng rice oil poisonings with polychlorinated biphenyls (PCBs) and dibenzofurans (PCDFs) in Japan (Masuda 2003) and Taiwan (Guo et al. 2003), respectively; Irish pork and produce contamination with dioxins (Tlustos et al. 2005; White and Birnbaum 2009); and dioxin contamination of Vietnamese food from Agent Orange (Schecter et al. 2003a, 2003b). Most episodes of POPs contamination in food are not well known.

Contamination of butter with POPs has been reported in Germany, Australia, and Egypt (Santillo et al. 2003). A worldwide survey of polychlorinated dibenzo-*p*-dioxins (PCDDs), PCDFs, PCBs, hexachlorobenzene, and 2,2-bis (4-chlorophenyl)-1,1,1-trichloroethane (DDT) in butter exists (Weiss et al. 2005). Wide variations in concentrations were found between and within regions. Weiss et al. (2005) suggested that butter was a suitable matrix for assessing POPs contamination of dairy products with POPs. Butter was reported as contaminated with PCDD/Fs in Germany from contaminated citrus pulp feedstuff from Brazil (Malisch 2000; Malisch et al. 2000; Muller et al. 2001). Butter in seven Egyptian provinces was reported to have a wide range of PCDD/F contamination (Malisch and Saad 1996).

Polybrominated diphenyl ether (PBDE) brominated flame retardants were not commonly used before the 1970s (Birnbaum and Staskal 2004), are considered primarily indoor contaminants (Allen et al. 2008), and are ingested or inhaled from food or dust (Bakker et al. 2008; Fischer et al. 2006; Harrad et al. 2008; Wu et al. 2007). Percent intake from food and dust sources is not well characterized and varies under differing conditions. Infants consume their mother’s milk, which is high in these fat-soluble compounds (Schecter et al. 2003c). Dust ingestion and inhalation become more important sources of exposure when children begin crawling on indoor and outdoor surfaces (Jones-Otazo et al. 2005; Wilford et al. 2005). Later in life, food and dust may each play a major role in intake. To the best of our knowledge, this is the first report of U.S. butter contaminated with PBDEs. This suggests that screening for toxic chemicals in food can reveal their presence in U.S. food and illustrates a potential route of exposure. Such screening would help reduce the incidence of U.S. food contamination and the amount of contaminated consumed food.

## Materials and Methods

### Sample collection

Ten samples of different brands of butter were collected from five supermarkets in Dallas, Texas, on two separate occasions in 2009. Samples were frozen at −80°C and shipped on dry ice to Eurofins laboratory in Germany. Equal proportions of the butter samples were combined to form a composite butter sample (Schecter et al. 2010c). We pooled samples to allow more types of POPs and a larger number of food types to be analyzed (Schecter et al. 2010a, 2010b, 2010c). The study showed elevated levels of PBDEs in the butter pool compared with our previous measurements. This led us to conduct individual sample analyses of the 10 butter samples. One sample contained significantly elevated levels of higher-brominated congeners compared with the other nine samples. By chance, the wrapping paper from this sample had not been discarded and was available for analysis.

### Chemical analysis

Measurement of PBDE levels in U.S. food has been described previously (Papke et al. 2004; Schecter et al. 2004, 2010c). All analyses were performed using the isotope dilution method. For each group of samples (6–10 samples), two quality control pools (one fishmeal and one butter sample) and a laboratory blank were analyzed in parallel.

Measurements were performed using high-resolution gas chromatography (HRGC)/high-resolution mass spectrometry (HP 5890 coupled with VG Autospec [Micromass Co. UK Ltd. (now Waters), Manchester, UK] at resolution point = 10,000 using a polyphenylmethylsiloxan 5 (30 m, 0.25 mm i.d., 0.1-μm film) column. Identification of brominated diphenyl ethers (BDEs) was based on retention time and isotope ratio. Quantification was performed using a five-point calibration curve. A number of samples were additionally analyzed using HRGC/low-resolution mass spectrometry with negative chemical ionization mode and DB 5 (15 m, 0.25 mm i.d., 0.1-μm film) column.

Solvents and reagents were tested for contamination before laboratory use. Glassware was rinsed with solvents prior to use. Silica gel and sodium sulfate were prewashed. To reduce risk of contamination, rotary evaporators were not used. No plastic equipment was used. Quantification was conducted only if sample data were at least twice the blank value.

Eurofins successfully participated in a number of international laboratory comparison studies measuring PBDEs in biological samples, including the Norwegian Institute of Public Health and Quality Assurance of Information for Marine Environmental Monitoring in Europe.

### Dietary intake estimation

Estimates of dietary intake have been described previously (Schecter et al. 2010c). The 2007 U.S. Department of Agriculture food availability data were employed to ensure use of the most recent dietary estimates.

## Results

Table 1 presents the congener-specific data for the 10 butter samples and wrapping paper that surrounded the highly contaminated stick of butter. In the highly contaminated sample, number 10, octabrominated (BDE-197), nonabrominated (BDE-206 and -207) and decabrominated (BDE-209) congeners were found at very high levels compared with the other butter samples. Sample 10 was not found to be contaminated above background with lower brominated congeners typically associated with the penta commercial mixture (BDE-47, -99, and -100). These were found to be below the median value of the 10 samples. The geometric means (GMs) for BDE-47 and -99 were 113 and 77.2 pg/g wet weight (ww), respectively, for all samples.

Butter sample 2 also contained higher levels of some congeners than found in the other butter samples. BDE-28, -47, -99, -100, -153, and -154 were elevated at levels of 7.6, 472.0, 489.0, 83.8, 72.0, and 24.4 pg/g ww, respectively, which are up to approximately four-fold higher than in the other samples.

Because of the markedly elevated levels of higher-brominated PBDE congeners measured in sample 10, we measured the levels of PBDEs in the sample 10 wrapper, which by chance was saved and available for analysis. This was not the case for the other butter samples. Levels of BDE-206, -207, and -209 in butter sample 10 were 2,000, 2,290, and 37,600 pg/g ww, respectively. Higher levels of these congeners were found in the sample 10 wrapper. Similar to the butter sample, BDE-209 was detected at the highest level (614,000 pg/g ww). The nonabrominated congeners BDE-206 and -207 were also detected at elevated levels (51,000 and 11,700 pg/g ww, respectively). The wrapper did not show any contamination with lower-brominated congeners, suggesting contamination from the deca-BDE commercial mixture. However, deca-BDE commercial mixture has been reported to have < 3% nona-BDE contamination. Our detected levels of BDE-206 and -207 total 4,020 pg/g ww, suggesting breakdown of BDE-209 in the commercial deca-BDE mixture.

Recalculation omitting butter sample 10 changes our previous estimate of total PBDE daily dietary intake of 50,386 pg/day to a new lower-bound level of 22,764 pg/day, with zero being used when the PBDE level was below the limit of detection (Table 2).

When values below the limit of detection (LOD) are treated as one half instead of zero (upper-bound estimation), the total estimated PBDE intake is 31,836 pg/day, with dairy and eggs contributing the largest amount of 11,014 pg/day. The updated percent of dietary intake of PBDEs in meat, fish, dairy and eggs, and vegetable products excluding the outlier changed from 18% to 40%, 3% to 7%, 77% to 48%, and 2% to 5%, respectively.

## Discussion

Here we describe the first known incidence of PBDE contamination of U.S. butter. The type of contamination in butter sample 10, which resembled commercial deca-BDE pattern, is most likely from the paper wrapper contamination with octa- and deca-BDE, either from electronic devices in the butter-processing plant or from contamination of the paper prior to delivery. The company has investigated the source of contamination and informed the authors that the company was unable to identify the source. Although the route of contamination remains unknown at this time, the congener distribution in the butter wrapper suggests contamination with the deca-BDE commercial mixtures, including evidence of some breakdown of the congeners found in deca-BDE to lower-brominated congeners. It is possible that the elevated levels of BDE-206 and -207 are due to debromination of BDE-209. The values measured in the butter sample for BDE-206, -207, and -209 ranged from 2% to 6% of the value measured in the wrapper, whereas for the octabrominated congeners BDE-196 and -197, the butter value was < 1% of the wrapper value. Similarly, BDE-183, a heptabrominated congener, had elevated levels in the butter wrapper (900 pg/g ww) but nondetectable levels in the butter sample. None of the other butter samples had detectable levels of BDE-209, suggesting that the contamination was solely from the wrapper. Additionally, many of the butter samples exhibited a higher level of BDE-47 compared with BDE-99, which is not observed in the penta-BDE commercial mixtures (La Guardia et al. 2006). These findings suggest that the components of the penta-BDE mixture are undergoing differential metabolism and/or biomagnification up the food chain.

Elevated levels of PBDEs from the penta-BDE commercial mixture were observed in butter sample 2 (4-fold compared with samples 1 and 3–9). However, because only BDE-47, -99, and -153 were elevated in both butter samples 2 and 10, it is possible that separate incidents of contamination occurred either for each separate butter sample and/or wrapper. Because the paper wrapper for butter sample 2 was not available for testing, we are uncertain of the source of contamination for this sample (butter itself vs. paper wrapper). Testing of all butter wrappers would have been desirable to help identify the source of contamination, but only the wrapper for sample 10 was available. However, we believe that this elevation of congeners from the penta-BDE commercial mixture is not indicative of special contamination, but instead is more likely to indicate variation of PBDE levels found in butter. We previously published findings of PBDE levels in human breast milk from the general population (Schecter et al. 2003c). BDE-47 showed up to an approximately 93-fold difference between highest and lowest levels in milk samples and up to a 15-fold difference between highest and median levels. Nevertheless, such elevations are worthy of consideration in future studies of U.S. butter and possibly represent special contamination with commercial penta-BDE mixture rather than a chance finding.

All samples had detectable levels of BDE-47, -99, and -100, and most contained detectable levels of BDE-153, suggesting possible contamination of the butter itself rather than from wrapping materials. Contamination may have occurred during the process of butter manufacture or, more likely, from contamination of the cow’s milk either after collection of milk from the cow or directly from the cow as a result of animal exposure to PBDEs through food and/or environment. Only butter sample 10 and its wrapper were contaminated with BDE-196, -197, -206, -207, and -209. Because these levels were extremely elevated, and because this contamination occurred only with this sample and its wrapper, it is likely that the wrapper was contaminated during production and subsequently contaminated the butter upon packaging.

When omitting the outlier, our new total daily PBDE dietary intake of 22,764 pg/day is lower than previously reported. Previous U.S. dietary intake estimates range approximately from 60,830 to 91,000 pg/day (Schecter et al. 2006, 2010b). Except for our most recent PBDE food survey publication, we performed our calculations using one-half the LOD when the findings were below that level. Here, we calculated using both zero and half the LOD value as lower- and upper-bound estimates when the PBDE level was below the LOD, as is commonly done when calculating exposure to PBDEs and related POPs (Huwe and Larsen 2005; Imm et al. 2009; Schecter et al. 2010b). Our upper-bound value for total PBDE food intake is 31,836 pg/day and our lower-bound limit is 22,764 pg/day. Omitting the outlier butter sample 10 in our calculations and using zero LOD yields these new lower values. However, even recalculating dietary intake without the contaminated butter sample, meat, dairy, and egg consumption still accounts for nearly 90% of PBDE dietary exposure. Other research groups from various countries have reported dietary PBDE intake higher than we report here. A 2004 dietary intake paper from the United Kingdom reported 90,500 pg/day, with levels below the LOD estimated as zero (Harrad et al. 2004). Dietary intake for Spain was reported approximately as 75,400 and 97,300 pg/day, estimating levels below the LOD at one-half (Bocio et al. 2003; Domingo et al. 2008). Estimates from Sweden and Finland were reported as approximately 51,000 and 44,000 pg/day, respectively, with values below the LOD calculated as half the LOD (Darnerud et al. 2001; Kiviranta et al. 2004). A study of PBDE contamination of U.S. cat food found elevated levels of BDE-209 and detectable levels of BDE-206 and -207 in all dry cat foods sampled, suggesting contamination with the deca-BDE commercial mixture (Dye et al. 2007). Wet cat foods were not found to be contaminated with such high levels of nona- and deca-BDEs but instead with penta-BDE congeners. This suggests that the preprocessed food materials themselves likely were not contaminated with the deca-BDE mixture, but that the contamination may have occurred during processing and packaging as with this study. In a previous study of PBDEs in U.S. foods, we found elevated levels of BDE-209 in cream cheese (481 pg/g ww) while no other dairy-based foods had levels > 20 pg/g ww (Schecter et al. 2006). We did not measure the cream cheese wrapper for PBDE contamination in that study; however, these earlier findings and this study suggest that food packaging could be an important route of PBDE contamination in a small subset of American foods.

The 64th report of the Joint FAO/WHO Expert Committee on Food Additives (JECFA 2007) reported dietary intake estimates of PBDEs in U.S. and Canadian diets as 213 ng/day total, with dairy products contributing 24 ng/day, eggs contributing 8 ng/day, fats and oils contributing 47 ng/day, fish and shellfish contributing 40 ng/day, meat and poultry contributing 66 ng/day, and other foods contributing 29 ng/day. These values are higher than our lower-bound estimate of dietary intake of PBDEs of 22.76 ng/day. This may be attributed partly to the fact that the JECFA North American estimated intakes include Canadian intakes. Regional variations in the United States may also contribute to the higher estimated intake (JECFA et al. 2007).

Our study also suggests that dietary exposure to PBDEs is higher than estimated indoor dust and water ingestion exposure. Lorber (2007) suggested that total PBDE adult intake of indoor dust via inhalation and water ingestion was estimated to be 5.5 and 0.2 ng/day, respectively (BDE-28, -47, -99, -100, -138, -153, -154, -183, and -209). Adult exposure via soil/dust ingestion and soil/dust dermal contact was estimated at 357.3 and 85.9 ng/day, respectively, higher than our lower-bound level of 22.76 ng/day (Lorber 2007).

Although the sample size is small, this study can serve to alert the public, scientists, food processors, and regulatory agencies that relatively high levels of food contamination with emerging POPs sometimes occurs. Although an exact source is not yet clear, we suspect that high levels of contamination can occur during food processing and packaging.

## Conclusions

The extent of food contamination with PBDEs and other emerging POPs in the United States is not well characterized. To protect the public health, carefully constructed, larger, representative studies of the U.S. food supply are indicated. Currently, the U.S. Food and Drug Administration does not have tolerances, guidance levels, or action levels established for PBDEs. Spot checks by regulatory agencies could help characterize the nature and extent of such potential health hazards. By investigating the circumstances under which chemical contamination of U.S. food occurs, it would be possible to determine when and where screening for POPs contamination of food is most appropriate and would also help reduce incidence of contaminated food sold to the public.

## Figures and Tables

**Table 1:** PBDE concentrations in 10 butter samples and one butter wrapper (pg/g ww).^a^

BDE congener	Sample 1	Sample 2	Sample 3	Sample 4	Sample 5	Sample 6	Sample 7	Sample 8	Sample 9	Sample 10	Sample 10 wrapper	Sample GMs	Sample medians
BDE-17	(5.8)	(1.45)	(0.8)	(6.3)	(0.9)	(0.6)	(0.7)	(0.5)	(1.5)	(1.5)	(50)	0.7	0.6
BDE-28	(5.1)	7.6	15.3	(5.5)	(0.8)	2.80	(1.4)	2.1	(2.4)	(2.4)	(50)	2.1	2.3
BDE-47	62.0	472.0	151.0	136.0	79.2	55.2	88.8	138.0	104.0	101.0	(100)	113	102.5
BDE-49	(1.8)	(2.3)	(1.5)	(3.1)	(1.6)	(1.6)	(1.4)	(1.2)	(1.9)	(2.7)	(100)	0.9	0.9
BDE-66	(1.9)	(2.4)	(1.6)	(3.3)	(1.6)	(1.7)	(1.4)	(1.3)	(1.9)	(2.7)	(100)	0.9	0.9
BDE-71	(1.8)	(2.4)	(1.6)	(3.2)	(1.6)	(1.7)	(1.4)	(1.3)	(1.8)	(2.7)	(100)	0.9	0.9
BDE-77	(1.1)	(1.4)	(0.9)	(1.8)	(0.9)	(0.9)	(0.8)	(0.7)	(1.0)	(1.4)	(100)	0.5	0.5
BDE-85	(1.4)	(5.4)	(3.4)	(8.4)	(2.6)	(2.2)	(1.7)	(1.7)	(4.9)	(4.5)	(100)	1.5	1.5
BDE-99	41.2	489.0	84.0	56.3	47.8	40.0	76.1	107.0	75.0	67.2	(100)	77.2	71.1
BDE-100	8.6	83.8	22.0	13.7	9.5	6.7	14.4	22.2	17.6	12.0	(100)	15.7	14.1
BDE-119	(1.5)	(5.7)	(3.6)	(9.0)	(2.8)	(2.4)	(1.8)	(1.8)	(5.3)	(4.9)	(100)	1.7	1.6
BDE-126	(0.9)	(3.4)	(2.2)	(5.4)	(1.7)	(1.4)	(1.1)	(1.1)	(3.1)	(2.9)	(100)	1.0	1.0
BDE-138	(4.5)	(5.2)	(6.6)	(13.7)	(3.6)	(2.0)	(4.8)	(4.2)	(4.3)	(4.4)	(200)	2.4	2.2
BDE-153	10.2	72.0	18.1	(14.0)	11.2	6.5	13.1	17.1	11.7	8.8	(200)	13.2	11.5
BDE-154	(3.5)	24.4	(5.2)	(10.7)	4.5	(1.5)	(3.8)	7.2	(3.6)	(3.7)	(200)	3.1	2.3
BDE-156	(5.4)	(6.3)	(8.0)	(16.6)	(4.4)	(2.4)	(5.8)	(5.0)	(5.1)	(5.3)	(200)	2.9	2.7
BDE-183	(5.7)	(7.7)	(9.4)	(3.3)	(3.8)	(2.7)	(3.1)	(4.9)	(6.2)	(4.7)	900	2.4	2.4
BDE-184	(5.3)	(7.2)	(8.8)	(3.1)	(3.6)	(2.6)	(2.9)	(4.6)	(4.6)	(4.4)	(300)	2.2	2.3
BDE-191	(4.0)	(5.4)	(6.6)	(2.3)	(2.7)	(1.9)	(2.2)	(3.4)	(3.2)	(3.3)	(300)	1.6	1.6
BDE-196	(9.2)	(9.6)	(9.2)	(8.1)	(6.2)	(7.1)	(4.8)	(6.8)	(8.3)	101.0	11,900	5.2	4.1
BDE-197	(9.5)	(9.9)	(9.5)	(8.3)	(6.4)	(7.3)	(5.0)	(7.0)	(8.6)	47.1	9,950	5.0	4.2
BDE-206	(9.9)	(7.2)	(6.6)	(7.5)	(10.3)	(4.9)	(8.4)	(7.3)	(8.0)	2,000	51,000	7.1	3.9
BDE-207	(7.4)	(5.4)	(13.2)	(5.6)	(7.7)	(3.6)	(6.3)	(5.5)	(10.8)	2,290	11,700	6.5	3.4
BDE-209	(31.5)	(39.8)	(32.2)	(30.4)	(43.7)	(32.9)	(37.8)	(48.7)	(51.6)	37,600	614,000	40.6	19.4
Lower-bound total BDE	122	1,148	290	206	152	111	192	294	208	42215.1	699,450	308	258
Upper-bound total BDE	180	1,212	355	290	205	151	240	347	277	42,252	804,751	483	284

a Samples below the limit of detection denoted with the detection limit in parentheses.

**Table 2:** Previous^a^ upper-bound^b^ and current^c^ lower- and upper-bound^b^,^d^ estimated adult dietary intake of PBDEs (pg/day).

	Meat			Fish			Dairy and eggs			Vegetable products			Total		
BDE total	Previous UBD	Current LBD	Current UBD	Previous UBD	Current LBD	Current UBD	Previous UBD	Current LBD	Current UBD	Previous UBD	Current LBD	Current UBD	Previous UBD	Current LBD	Current UBD
Total tri-BDE	56	56	67	63	63	79	270	260	52	1	1	102	391	380	300

Total tetra-BDE	1,826	1,826	1,871	1,021	1,021	1,026	2,543	2,401	2,499	558	558	847	5,948	5,806	6,243

Total penta-BDE	2,408	2,408	2,489	336	336	342	2,303	2,088	2,601	479	479	4,099	5,525	5,310	9,530

Total hexa-BDE	667	667	727	105	105	115	396	394	733	5	5	644	1,174	1,172	2,219

Total hepta-BDE	756	756	1,195	4	4	19	44	44	192	0	0	514	804	804	1,921

Total octa-BDE	424	424	559	1	1	10	239	118	128	0	0	341	665	544	1,037

Total nona-BDE	386	386	501	8	8	19	3,774	1,035	498	0	0	690	4,167	1,428	1,709

Total deca-BDE	2,575	2,575	2,743	58	58	99	29,079	4,686	4,311	0	0	1,724	31,713	7,320	8,878

Total PBDEs	9,099	9,099	10,152	1,595	1,595	1,709	3,8648	11,027	11,014	1,043	1,043	8,961	50,386	22,764	31,836

a Previous estimates from Schecter et al. (2010c).

b Estimate setting limit of detection = one-half refers to upper-bound estimate (UBD).

c Current estimates based on samples from previous estimate (Schecter et. al. 2010c) excluding butter sample 10.

d Estimate setting limit of detection = 0 refers to lower-bound estimate (LBD).
